# Exploring Symptoms of Borderline Personality Disorder in People With Affective Disorders: A Network Analysis

**DOI:** 10.31083/AP47292

**Published:** 2025-10-13

**Authors:** Hyeona Yu, Youkyung Hwangbo, Daseul Lee, Jakyung Lee, Yuna Kim, Chan Woo Lee, Hyukjun Lee, Yoonjeong Jang, Junwoo Jang, Hyo Shin Kang, Ji Hyun Baek, Tae Hyon Ha, Jungkyu Park, Woojae Myung

**Affiliations:** ^1^Department of Neuropsychiatry, Seoul National University Bundang Hospital, 13620 Seongnam, Republic of Korea; ^2^Department of Psychology, Kyungpook National University, 41566 Daegu, Republic of Korea; ^3^Department of Psychiatry, School of Medicine, Samsung Medical Center, Sungkyunkwan University, 06351 Seoul, Republic of Korea; ^4^Department of Psychiatry, Seoul National University College of Medicine, 03080 Seoul, Republic of Korea

**Keywords:** affective disorders, major depressive disorder, bipolar disorder, network analysis, borderline personality disorder symptoms

## Abstract

**Objective:**

Borderline personality disorder (BPD) frequently co-occurs with affective disorders, such as major depressive disorder (MDD) and bipolar disorder (BD), yet the structure of BPD symptoms within these populations remains insufficiently characterized. This study utilizes network analysis to investigate the network structure of BPD symptoms in individuals with affective disorders.

**Methods:**

This study included 1323 participants: 783 individuals with affective disorders (MDD [n = 245], BD I [n = 120], BD II [n = 418]) and 540 controls without a history of psychiatric disorders. BPD symptoms were assessed using the Personality Assessment Inventory-Borderline Features Scale. A Gaussian graphical model was estimated using partial correlations among BPD symptoms, in which nodes correspond to individual symptoms and edges represent the relationships between them. Centrality analysis was subsequently conducted to compute strength, closeness, and betweenness centrality, providing insights into the relative importance and connectivity of individual symptoms within the network.

**Results:**

Analysis identified five distinct communities of BPD symptoms. The symptom “Feel empty” emerged as the most central trait across the affective disorder subgroups and the control group. Network comparison tests indicated no significant differences in network structure among the clinical subgroups, whereas a significant divergence was observed between the clinical and control groups.

**Conclusion:**

This study demonstrates that, although the symptom networks of BPD were largely comparable across affective disorder subgroups, structural differences emerged between the clinical and control groups. Notably, “Feel empty” consistently appeared as the most central symptom across all groups. These findings highlight the relevance of targeting “Feel empty” as a key focus for clinical intervention in affective disorders.

## Main Points

1. Borderline personality disorder (BPD) symptoms in individuals with affective disorders were organized into five distinct symptom communities.

2. “Feel empty” consistently emerged as the most central symptom across all groups.

3. A significant structural difference in BPD symptom networks was observed between the clinical and control groups.

## 1. Introduction

Borderline personality disorder (BPD) is characterized by pathological symptoms such as negative affectivity, identity disturbance, negative interpersonal relationships, and impulsive behaviors [1]. BPD symptoms often involves self-harm or impulsive behaviors, rapid mood changes, and unstable relationships [2]. Some studies have found that symptoms of BPD, such as feelings of emptiness and intense sadness [3, 4], often overlap with those of affective disorders, including major depressive disorder (MDD) and bipolar disorder (BD). Additionally, shared symptoms of BPD and affective disorders, such as negative affectivity and cyclothymic temperament, can increase the risk of misdiagnosis, making it difficult to distinguish between them [5]. These findings highlight the need for further research on the role of BPD symptoms in relation to affective disorders.

Although BPD symptoms and affective disorders share common symptoms, and their comorbidity is widely reported in the literature [6, 7, 8], prior study have primarily focused on BPD as a categorical diagnosis rather than examining BPD symptoms dimensionally. Consequently, while BPD is extensively studied in relation to affective disorders, fewer study investigate their structure, interconnectedness, and correlations in individuals experiencing MDD and BDs. This creates a critical gap in understanding how specific BPD symptoms interact and influence one another in individuals with affective disorders.

Research largely focuses on comparing the overall severity of BPD symptoms in individuals with affective disorders rather than analyzing its network structure. Although these study provide valuable insights, they fail to capture the complex interplay and correlations between individual BPD symptoms and how these interactions contribute to clinical presentations in different affective disorder subgroups. Furthermore, research on the interconnectedness and correlation of core BPD symptoms in individuals with affective disorders remains scarce.

To address this gap, this study employs network analysis, a recent psychiatric research approach intended for examining the relationships between entities. This method allows us to identify central symptoms, called nodes, and their connection between disorders, called edges [4, 9]. Critically, network analysis goes beyond simply identifying individual symptoms and their severity to explore their relationships. It visualizes and ranks the strength of the connections or interconnections between symptoms [10], revealing their complex interplay and correlations. This approach enables the exploration of both prominent symptoms and their interconnectedness within and across MDD, BD, and a control group. It has proven to be a powerful tool for identifying comorbidity and bridge symptoms for targeted intervention, providing insights into how symptoms are associated and suggesting intervention targets for effective treatment [4].

This study primarily aims to investigate the network structure of BPD symptoms in individuals with affective disorders. Specifically, it investigates the central symptoms of BPD and identifies distinct communities of interconnected symptoms of borderline personality disorder within an affective disorder population. Furthermore, the study seeks to compare the network structure of BPD symptoms across different subgroups of affective disorders (i.e., MDD, BD I, and BD II) and a control group.

## 2. Materials and Methods

### 2.1 Participants

A total of 1323 participants were recruited for this study between September 2019 and January 2023, ranging in age from 16 to 69 years. Among the participants, 783 participants were diagnosed with affective disorders by certified psychiatrists (MDD [n = 245], BD I [n = 120], and BD II [n = 418]) and the remaining 540 participants reported no history of psychiatric disorders. affective disorder diagnoses were determined through structured diagnostic interviews conducted by certified psychiatrists (J.H.B, T.H.H, and W.M.). The interviews were performed using the Mini-International Neuropsychiatric Interview (M.I.N.I) as well as the review of case records and relevant data (e.g., psychiatric interview records, clinical psychological assessments, and medical records from other hospitals provided by the subjects) [11]. These evaluations were conducted in accordance with the criteria outlined in the Diagnostic and Statistical Manual of Mental Disorders, Fifth Edition (DSM-5) [12]. Demographic information and BPD symptoms profiles were also collected from the study participants for further analysis. The control group consisted of individuals recruited through a professional survey company, with inclusion criteria requiring participants to self-report no current or past psychiatric diagnoses. This group serves as a baseline for comparison, helping to identify network structural differences observed between clinical and control groups, isolating those attributable to borderline personality disorder symptoms. This study was conducted under the guidelines of the Declaration of Helsinki (1975, as revised in 2000) and received ethical approval from the Institutional Review Board of Seoul National University Bundang Hospital (protocol code: B-2205-756-111). Given the retrospective nature of this study and the use of de-identified data, the requirement for individual informed consent was waived. Participants with lived experience were not directly involved in this study, as the research relied solely on de-identified data obtained through retrospective medical chart reviews and anonymized surveys. The reporting of our observational study followed the Strengthening the Reporting of Observational Studies in Epidemiology (STROBE) cross-sectional checklist (The STROBE cross-sectional checklist is provided in the **Supplementary material-STROBE-checklist**.) [13].

### 2.2 Measurements

#### Personality Assessment Inventory-Borderline Features Scale (PAI-BOR)

The PAI-BOR, a measuring instrument for evaluating adult personality, is one of eleven clinical scales included in Morey’s (1991) Personality Assessment Inventory (PAI) [2]. It is a self-report questionnaire designed to assess key characteristics of BPD, such as emotional instability, identity problems, unfavorable interpersonal connections, and propensity for self-harm. The scale used in this investigation is a validated Korean version [14], having 24 items, each of which is scored on a 4-point scale (0 = never true to 3 = very true). The total score ranges from 0 to 72, with higher scores indicating a greater severity of borderline personality disorder symptoms. The PAI-BOR comprised 24 items that assessed symptoms of borderline personality disorder across four subscales: (1) Affective Instability (e.g., mood change, extreme feelings, sudden emotional shifts, and mood swings), (2) Identity Problems (e.g., attitude change, emptiness; chronic feelings of emptiness, and uncertainty about self-image), (3) Negative Relationships (e.g., interpersonal instability, a few major mistakes with friends; intense and unstable interpersonal relationships characterized by distrust and dependency), and (4) Self-Harm/Impulsivity (e.g., impulsive behavior, “When I am angry, I usually harm myself”; impulsive behaviors and tendencies toward self-harm). These symptom dimensions were evaluated using all 24 items of the PAI-BOR to provide a comprehensive assessment of BPD symptoms at the item level. This item-level approach enabled a detailed analysis of specific symptoms and their interconnections within the network. The internal consistency coefficient (Cronbach’s alpha), which was reported to be 0.84 in a prior study [14], was 0.83 in this study.

### 2.3 Statistical Analysis

#### 2.3.1 Comparison of PAI-BOR Item Scores Among Clinical and Control Groups

The Shapiro-Wilk test was conducted to assess the normality of the PAI-BOR item scores. Since the scores did not follow a normal distribution, the Kruskal-Wallis test, a non-parametric statistical method, was utilized to compare the scores among each clinical group (MDD, BD I, and BD II) and the control group.

#### 2.3.2 Network Estimation With Community Detection Process

The network analysis utilized all 24 PAI-BOR items rather than aggregating them into subscales (e.g., negative affectivity and identity disturbance). This approach was chosen to preserve item-level granularity, enabling a detailed examination of symptom interconnections and community structures. Analyzing individual items enables the identification of central symptoms and the detection of distinct symptom clusters through exploratory graph analysis. This study employed a Gaussian graphical model (GGM) to construct a Partial Correlation Network (PCN) [15]. The PCN comprises circular nodes and line-shaped edges. In this representation, nodes represent symptoms and edges depict connections between these symptoms [16]. Specifically, each edge reflects a partial correlation between two symptoms, adjusting for all other symptoms within the network [17]. The color of the lines denotes the direction of associations between edges: a blue and red line indicates a positive and negative correlation, respectively. Strength centrality indicates the extent to which a node is tightly connected to other nodes in the network by summing the absolute edge weights between the node and others. Betweenness centrality measures how often a node appears on the shortest paths between two nodes in the network. Closeness centrality reflects the distance a specific node needs to reach other nodes. The width of the edges illustrates the strength of the relationship, where substantial lines indicate stronger correlations and nodes positioned in closer proximity represent stronger associations with each other.

In PCN, the absence of an edge signifies a lack of significant association between the two symptoms, considering the associations with other symptoms. Nevertheless, it is unusual for the correlations between symptoms to be exactly zero in practice, given the presence of small partial correlations [15]. Such insignificant correlations can give rise to spurious edges, potentially leading to a misinterpretation of the associations in the network. To address this issue, we adopted a graphical Least Absolute Shrinkage and Selection Operator (LASSO) [18] which helps search for the optimal level of sparsity by reducing the insignificant partial correlations to zero. The study utilized the Extended Bayesian Information Criterion graphical lasso (EBICglasso) function from the qgraph package (version 1.9.5; Sacha Epskamp, University of Amsterdam, Amsterdam, Netherlands) to construct and visually represent a parsimonious and plausible network [18, 19].

In this study, we utilized exploratory graph analysis (EGA) to identify item communities within a generated network [20, 21]. EGA is a data-driven approach for detecting communities, aiming to uncover the underlying dimensional structure of given items without relying on prior knowledge about the structure [22]. To discover the optimal communities of the 24 items, we used the Louvain algorithm, which optimizes hierarchically organized modularity measure between vertices in item communities [23].

A bootstrapping procedure was employed to assess item and dimensional stability. We specifically re-estimated the item community assignments, specifying which item belonged to each item community and the number of dimensions, using 1000 bootstrapped samples to determine whether the solution previously obtained from the EGA was maintained [24]. The bootEGA and EGA functions from the EGAnet package version 2.0.4 (Hudson F. Golino, University of Virginia, Charlottesville, VA, USA; 2024) were used to perform these analyses [25].

#### 2.3.3 Centrality Estimates Analysis

To examine prominent BPD symptoms within the network, an analysis of three centrality indices was conducted, encompassing strength, closeness, and betweenness. Strength centrality was computed as the total of the absolute weights of the connected edges to a node, reflecting the extent to which the activation of a node is related to the activation of its connected nodes. Closeness centrality was computed as the reciprocal of the average shortest distance from one node to all other nodes in the network, demonstrating how easily a node can be reached from other nodes. The extent to which a node acts as a mediator in connecting two other nodes along its shortest path determines its betweenness centrality [26]. Centrality indices, including strength, closeness, and betweenness, were calculated using the R package qgraph (version 1.9.5) to assess the importance of each node within the network [27].

#### 2.3.4 Network Stability and Testing for Significance

To determine the accuracy and stability, we first calculated the 95% confidence intervals (CIs) for the edge weights using 1000 bootstrap resamples, where narrow bootstrapped CIs indicated small sample variability, supporting the accuracy of the network. Furthermore, we also assessed the robustness of node centrality by repeatedly analyzing the data after randomly excluding subsets of cases. This analysis measures how well centrality orders were preserved across multiple data. The correlation stability coefficient (CS-coefficient) summarizes the results of such case-dropping bootstrapping samples. A CS-coefficient of at least 0.25 is considered the minimum threshold, while a coefficient exceeding 0.5 indicates acceptable stability [18]. Additionally, we conducted bootstrapped difference tests to examine the differences in node centrality indices across different groups. We particularly conducted a bootstrapped difference test for strength centrality between the clinical and control groups, aiming to explore whether central symptoms exhibited notable disparities between these two groups. The qgraph package was employed for the analyses [27].

#### 2.3.5 Network Comparison

We conducted a Network Comparison Test (NCT) using the NCT package (version 2.2.1; Sacha Epskamp & Adela-Maria Isvoranu, University of Amsterdam, Amsterdam, Netherlands) to compare the network structure within each pair of clinical samples (i.e., MDD, BD I, and BD II) using the NCT package [28]. Three analyses were performed to compare different aspects of the networks. First, a test for invariance in the structural composition of the network was employed to evaluate disparities in its maximum edge strength. Second, the global strength invariance test was used to evaluate variations in total edge strengths. Third, an edge strength invariance test was used to investigate specific edge differences in the networks [28].

## 3. Results

### 3.1 Study Sample

The demographic features of the participants are presented in Table 1. This study included 1323 participants, of whom 783 were diagnosed with MDD, BD I, and BD II in the clinical group, and 540 were in the control group. Table 2 presents the differences in mean scores for PAI-BOR items between each clinical (MDD, BD I, and BD II) and control groups.

**Table 1.** **Demographic and clinical characteristics of research participants (N = 1323)**.

Characteristics		Mean ± SD, n or %				
Major diagnosis (n)		Control (540)	MDD (245)	BD I (120)	BD II (418)	Spearman’s rho or $\chi^{2}$
Age (years)		39.52 ± 10.92	39.73 ± 13.42	35.48 ± 12.67	31.14 ± 11.46	–0.31^*⁣**^
Age range (n)	16∼19	5	8	5	32	160.73^*⁣**^
	20∼29	130	66	48	206	
	30∼39	135	44	31	82	
	40∼49	135	61	12	55	
	50∼59	128	50	20	36	
	60∼69	7	16	4	7	
Gender n (%)	Male	270 (50.0)	69 (28.2)	43 (35.8)	119 (28.5)	59.50^*⁣**^
	Female	270 (50.0)	176 (71.8)	77 (64.2)	299 (71.5)	
Education n (%)	Less than high school grate	1 (0.2)	95 (38.8)	28 (23.3)	129 (30.9)	225.01^*⁣**^
	Others	539 (99.8)	150 (61.2)	92 (76.7)	289 (69.1)	
Job status n (%)	Unemployed	170 (31.5)	144 (58.8)	76 (63.3)	281 (67.2)	139.52^*⁣**^
	Employed	370 (68.5)	101 (41.2)	44 (36.7)	137 (32.8)	
Marital status n (%)	Married	255 (47.4)	132 (53.9)	82 (68.3)	306 (73.2)	77.52^*⁣**^
	Others	285 (52.6)	113 (46.1)	38 (31.7)	112 (26.8)	
Alcohol use status n (%)	Past or current	423 (78.3)	93 (38.0)	70 (58.3)	220 (52.6)	146.84^*⁣**^
	Never	117 (21.7)	152 (62.0)	50 (41.7)	198 (47.4)	
Smoking status n (%)	Past or current	188 (34.8)	49 (20.0)	35 (29.2)	130 (31.1)	17.68^*⁣**^
	Never	352 (65.2)	196 (80.0)	85 (70.8)	288 (68.9)	

^*⁣**^p < 0.001.  MDD, major depressive disorder; BD, bipolar disorder.

**Table 2.** **PAI-BOR item scores in clinical and control groups**.

Item description		Mean (SD)				Kruskal Wallis test	
		Control (n = 540)	MDD (n = 245)	BDI (n = 120)	BDII (n = 418)	H^‡^	Bonferroni post hoc^§^
1	Mood shifts	0.62 (0.76)	0.97 (0.89)	0.81 (0.90)	1.37 (1.02)	143.28^*⁣**^	0<1, 0<3, 1<3, 2<3
2	Attitude about self-changes	0.57 (0.72)	0.84 (0.86)	0.82 (0.87)	1.28 (1.03)	124.63^*⁣**^	0<1, 0<3, 1<3, 2<3
3	Relationships stormy	0.57 (0.79)	0.94 (1.03)	0.99 (1.09)	1.28 (1.11)	107.25^*⁣**^	0<1, 0<2, 0<3, 1<3
4	Mood intense	0.58 (0.76)	0.67 (0.87)	0.88 (0.96)	1.28 (1.08)	118.74^*⁣**^	0<2, 0<3, 1<3, 2<3
5	Feel empty	1.07 (0.84)	1.78 (0.99)	1.36 (1.08)	2.08 (0.99)	238.15^*⁣**^	0<1, 0<3, 1<3, 2<1, 2<3
6	Let people know they’re hurt me	0.69 (0.86)	1.03 (1.05)	1.01 (1.09)	1.42 (1.16)	101.15^*⁣**^	0<1, 0<2, 0<3, 1<3, 2<3
7	Mood steady^†^	1.47 (0.83)	2.33 (0.76)	1.75 (0.96)	2.44 (0.78)	320.34^*⁣**^	0<1, 0<2, 0<3, 2<1, 2<3
8	Worry about people leaving	0.77 (0.85)	0.88 (0.99)	1.02 (1.13)	1.41 (1.11)	85.04^*⁣**^	0<3, 1<3, 2<3
9	People let me down	0.96 (0.83)	1.30 (0.98)	1.27 (1.10)	1.54 (1.04)	75.52^*⁣**^	0<1, 0<2, 0<3, 1<3
10	Little control over anger	0.48 (0.74)	0.58 (0.78)	0.58 (0.90)	0.81 (0.93)	37.00^*⁣**^	0<3, 1<3
11	Wonder about life	1.53 (0.88)	1.73 (0.96)	1.55 (0.98)	2.06 (0.95)	80.66	0<3, 1<3, 2<3
12	Rarely lonely^†^	1.81 (0.91)	2.19 (0.89)	2.17 (0.87)	2.34 (0.93)	100.66^*⁣**^	0<1, 0<2, 0<3
13	Do things impulsively	0.51 (0.74)	0.55 (0.72)	0.83 (0.96)	1.02 (1.00)	80.23^*⁣**^	0<2, 0<3, 1<3
14	Happy person^†^	1.74 (0.85)	2.47 (0.73)	2.03 (0.86)	2.60 (0.65)	280.24^*⁣**^	0<1, 0<2, 0<3, 2<1, 2<3
15	Can’t handle separation	0.64 (0.78)	0.74 (0.81)	0.86 (1.00)	0.98 (0.99)	27.50^*⁣**^	0<3, 1<3
16	Mistakes in picking friends	0.73 (0.78)	0.54 (0.71)	0.90 (0.86)	0.86 (0.91)	24.32^*⁣**^	1<2, 1<3
17	When upset hurt self	0.49 (0.71)	0.71 (0.87)	0.70 (0.92)	1.10 (1.08)	85.37^*⁣**^	0<1, 0<3, 1<3, 2<3
18	Can’t express all of anger	0.82 (0.87)	1.28 (1.00)	1.09 (1.04)	1.43 (1.09)	84.89^*⁣**^	0<1, 0<3, 2<3
19	Don’t get bored^†^	1.84 (0.85)	1.95 (0.89)	2.10 (0.83)	2.30 (0.78)	74.53^*⁣**^	0<3, 1<3
20	Stay friends with people^†^	1.43 (0.79)	1.47 (0.90)	1.49 (0.93)	1.54 (0.96)	*NS*	-
21	Too impulsive	0.66 (0.77)	0.68 (0.79)	0.85 (0.98)	1.00 (0.95)	35.71^*⁣**^	0<3, 1<3
22	Spend money easily	0.82 (0.89)	0.87 (0.91)	1.28 (1.06)	1.42 (1.08)	90.98^*⁣**^	0<2, 0<3, 1<2, 1<3,
23	Reckless person	0.73 (0.80)	0.83 (0.81)	1.00 (0.95)	1.14 (1.02)	40.97^*⁣**^	0<3, 1<3
24	Careful about money^†^	1.26 (0.89)	1.42 (0.91)	1.37 (1.00)	1.43 (1.02)	9.68^*^	-
Total score		22.79 (10.13)	28.73 (10.86)	28.68 (13.56)	36.13 (12.84)	250.52^*⁣**^	0<1, 0<2, 0<3, 1<3, 2<3

^*^p < 0.05, ^*⁣**^p < 0.001, *NS* = Non-significant coefficient. ^†^ 7, 12, 14, 19, 20, 24 items reverse scored prior to analysis. ^‡^ Kruskal Wallis test. ^§^ Adjusted *p*-values were calculated using Bonferroni’s correction by multiplying the raw *p*-values by the total number of multiple tests of subscales.  0 = Control group, 1 = MDD, 2 = BD I, 3 = BD II.  PAI-BOR, Personality Assessment Inventory-Borderline Features Scale.

### 3.2 Network Construction and Community Identification

**Fig. 1.**
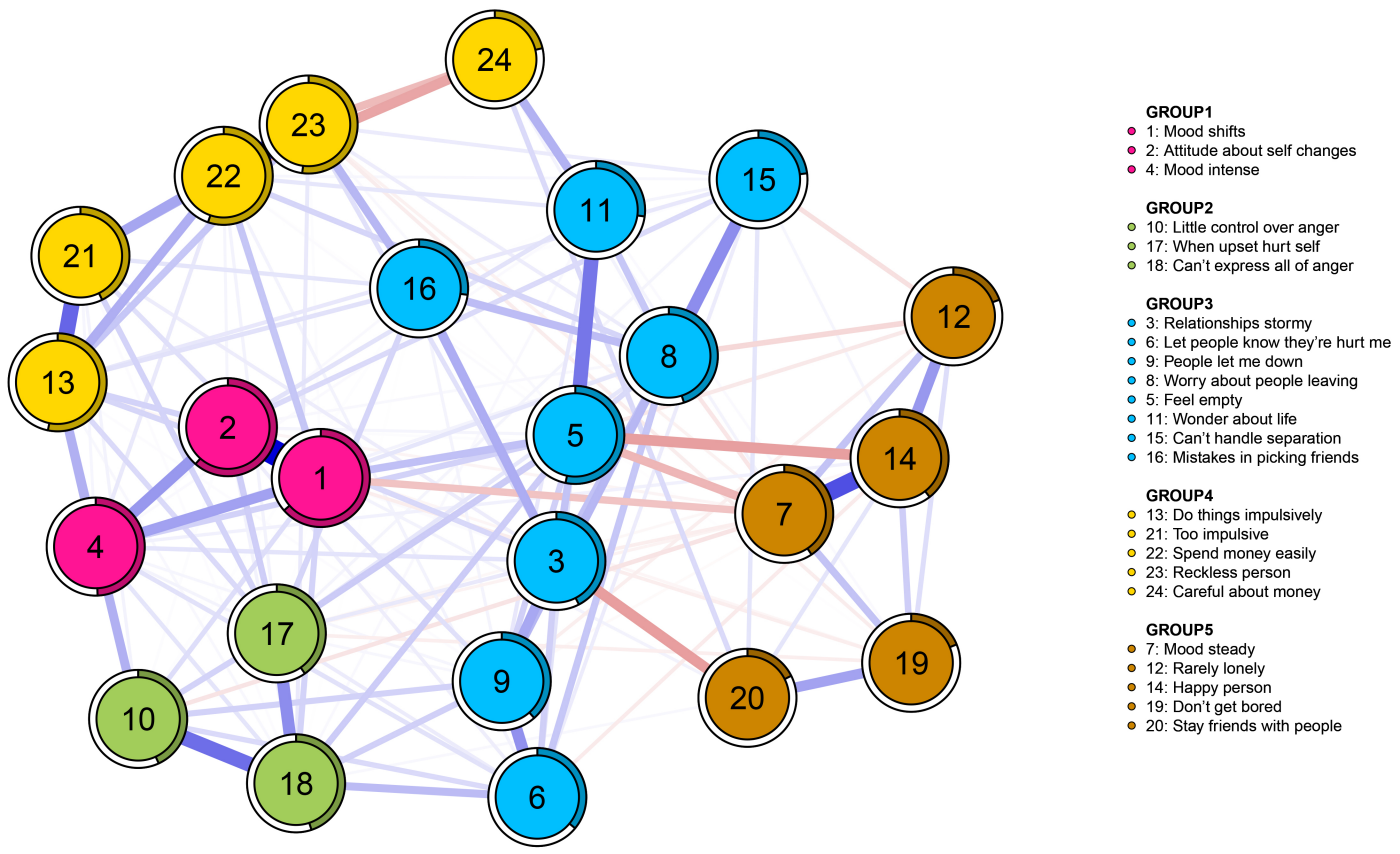
**Network of the PAI-BOR 24-symptom-items in subjects with affective disorder (n = 783)**. PAI-BOR, Personality Assessment Inventory-Borderline Features Scale.

Fig. 1 illustrates the network of BPD symptoms in subjects with affective disorders based on EGA. Community 1 comprises items 1, 2, and 4; Community 2 includes items 10, 17, and 18; Community 3 comprises items 3, 5, 6, 8, 9, 11, 15, and 16; Community 4 encompasses items 13, 21, 22, 23, and 24; and finally, Community 5 includes items 7, 12, 14, 19, and 20 (**Supplementary Fig. 1**). The results of bootstrap iterations aimed at examining dimensional stability showed that the five community solution exhibited 50.6% of the replication rate (506 of 1000 samples). Furthermore, the results of verifying the stability of each item (**Supplementary Fig. 2**) revealed that all items exhibited an acceptable level of stability, except for the following three items, which had an item replication rate of less than 0.7: items 11 (“Wonder about life”), 5 (“Feel empty”), and 20 (“Stay friends with people”) [24]. **Supplementary Fig. 3** depicts the network structure of BPD symptoms derived from the control group based on exploratory graph analysis (EGA). The results showed that Community 1 comprised items 1, 2, 4, 6, 10, and 13; Community 2 included items 21, 22, and 23; Community 3 consisted of items 3, 8, 9, 15, 16, 17, and 18; and finally, Community 4 comprised items 5, 7, 11, 12, 14, 19, 20, and 24 (**Supplementary Fig. 4**).

### 3.3 Centrality Indices and Edge Weights

**Fig. 2.**
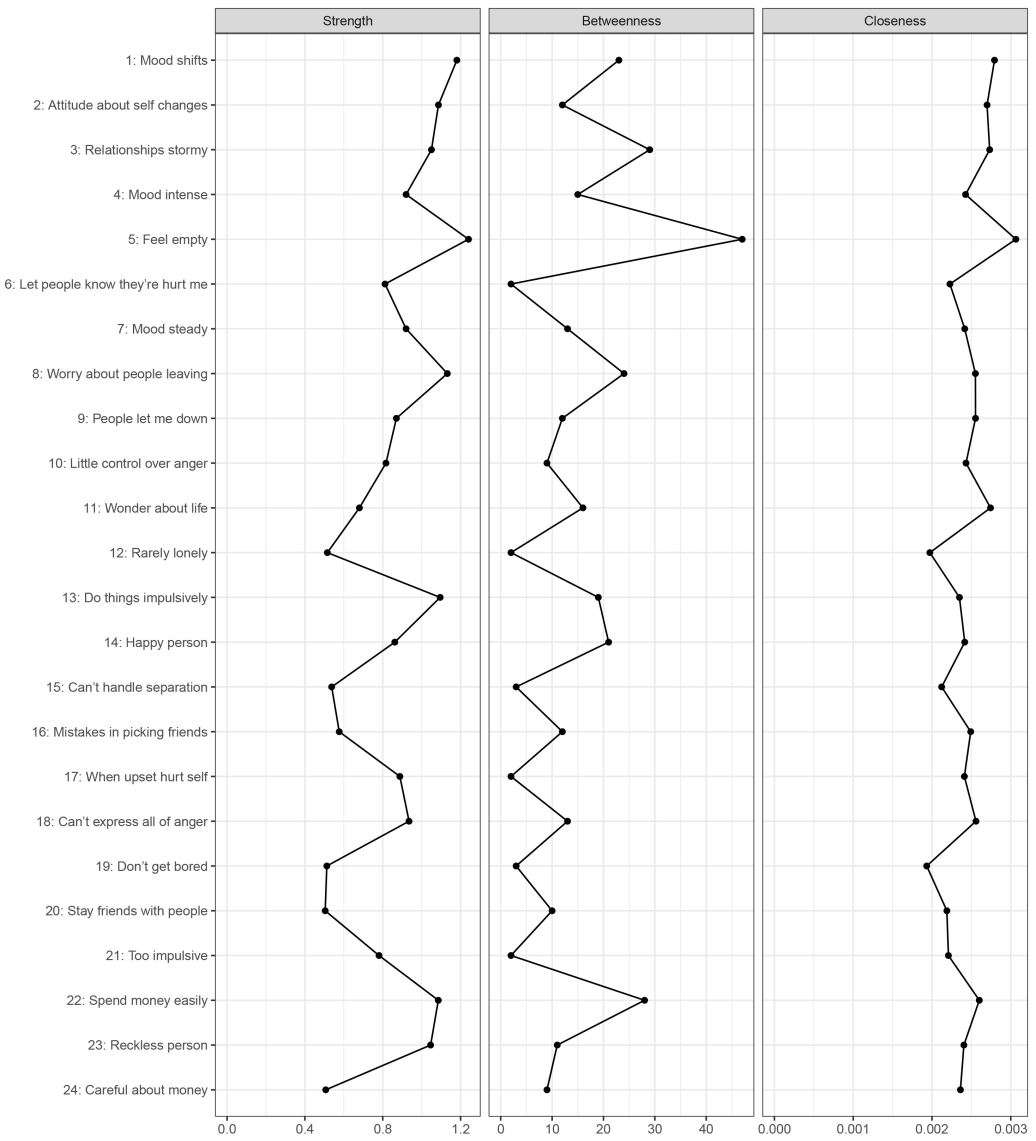
**Centrality indices of the PAI-BOR 24-symptom-items network**.

Fig. 2 illustrates these centrality indices. The results revealed that item 5 (“Feel empty”) demonstrated the highest centrality across all three centrality indices (strength = 1.240; betweenness = 94; closeness = 0.003). The strength centrality results remained consistent after performing the bootstrapped difference test (**Supplementary Fig. 5**).

### 3.4 Highest Centrality Item Exhibited by Affective Disorder Subgroups and Control Group

Additional analyses were conducted to examine whether item 5 (“Feel empty”), identified as the central node, was consistently replicated across all subgroups. The results revealed that item 5 (“Feel empty”) was the most central item in all subgroups’ networks, including each clinical sample (i.e., MDD, and BD I and BD II) and control group. An exception was item 1 (“Mood shifts”), which showed the highest centrality in each group with statistically nonsignificant difference from item 5 (**Supplementary Fig. 6–9**).

### 3.5 Network Stability

**Supplementary Fig. 10** illustrates the network stability indices (strength, betweenness, and closeness). Bootstrapped the centrality stability result revealed that the strength centrality index of BPD symptoms was the most stable (75%); and these results were maintained for other indices. Furthermore, the betweenness and closeness centrality results showed acceptable CS coefficients until 36.1% and 43.9% of the samples were removed, respectively, suggesting that both indices may be less reliable, even when the sample size is small.

Edge weight accuracy results show the robustness of the derived BPD symptom network** (Supplementary Fig. 11)**. Although there are no definitive guidelines for interpreting CIs in the network analysis literature [29] the results showed that the 95% CIs of the edge weights overlapped considerably. Considering that the bootstrapped CIs showed no intersection with those of other edges within the network, the edges between item 1 (“Mood shifts”) and item 2 (“Attitude about self-changes”) as well as item 22 (“Spend money easily”) and item 23 (“Reckless person”) were found to be the two strongest edges. The bootstrapped difference tests between edge weights provide additional supporting evidence, confirming that these two edges are significantly stronger than the others. Conversely, no significant difference was found between these two edges (**Supplementary Fig. 12)**.

### 3.6 Network Comparisons Test Between the Subgroups

Network comparison test confirmed that there were no differences in the network structure and edge weight in pairs of clinical samples (i.e., individuals diagnosed with MDD, BD I, and BD II) (MDD vs. BD I, M = 0.267, *p* = 0.978; MDD vs. BD II, M = 0.144, *p* = 0.891; BD I vs. BD II, M = 0.197, *p* = 0.986). Moreover, no statistical difference was found in the network structure between any pair of clinical subgroups, and no edges exhibited significant differences across clinical subgroups. Furthermore, among the clinical subgroups, global strength did not exhibit notable differences (MDD vs. BD I, S = 0.110, *p* = 0.999; MDD vs. BD II, S = 0.413, *p* = 0.447; BD I vs. BD II, S = 0.303, *p* = 0.984). Additionally, we compared the network structures of BPD symptoms between the clinical (MDD, BD I, and BD II combined) and control groups using a Network Comparison Test. The results showed significant structural differences (M = 0.187, *p* = 0.013) between the two groups, with stronger interconnections observed among symptoms in the clinical group. However, no significant difference was found in edge invariance and global strength (S = 0.185, *p* = 0.679).

## 4. Discussion

In this study, a network analysis was employed to explore the core symptoms of BPD in participants with affective disorders. The most central symptom identified through the analysis was “Feel empty,” which exhibited the highest node strength across all clinical groups, signifying its strong influence and interconnectedness with other symptoms within the network. This finding was further validated through bootstrapped difference tests and case-dropping procedures, which provided evidence supporting the stability and robustness of the node ranking.

In our investigation of the core BPD symptoms in individuals with affective disorders, we identified “Feel empty” as a pivotal symptom, revealing significant insights into the complexity of the disorder. This finding is particularly critical, as the comorbidity of this symptom with affective disorders remains underexplored in existing literature, especially through a network structure approach. The core symptom in this study, “Feel empty,” closely aligns with the concept of chronic emptiness, a fundamental aspect of BPD, as outlined in the DSM-5 alternative diagnostic model [30, 31]. This emotional state may be linked to social and self-perception difficulties, hindering the ability of an individual to form meaningful connections and potentially intensify feelings of emptiness [30, 31].

Our exploration into the network analysis of BPD symptoms, rather than solely focusing on diagnosed BPD, highlights the significant overlap between affective disorders and borderline personality disorder symptoms. This overlap may reflect shared vulnerabilities or overlapping symptoms rather than suggesting a direct progression from affective disorders to BPD. These findings underscore the importance of addressing core BPD symptoms in individuals with affective disorders to improve clinical outcomes. This method reveals the complex interplay between BPD symptoms and affective disorders through the identification of five distinct symptom communities, providing a more nuanced understanding of the BPD system beyond what factor analysis could offer. This comprehensive approach highlights the intricate relationships within these symptom communities, establishing a multifaceted perspective on comorbidity symptoms in BPD among affective disorders and its management within this context. This study’s findings deepen the understanding of BPD symptoms intricacies in individuals with affective disorders and also may lay the groundwork for future studies aimed at intervention and treatment strategies. By highlighting the common coexistence of BPD and affective disorders, our finding may underscore the importance of focusing on interventions for core symptoms before they develop into full BPD, advocating for a proactive treatment approach [10, 16, 30].

Borderline symptoms were the most severe in individuals with BD II compared to those in other groups (Table 2). This may be attributed to the chronic depressive episodes in BD II, which intensify core BPD symptoms, such as emotional instability and feelings of emptiness [31]. These features overlap with the cyclothymic temperament, a subclinical form of BD characterized by emotional reactivity and impulsivity [32]. Furthermore, BPD exacerbates BD symptoms such as suicidality and mood instability and highlights the heightened severity of BPD symptoms in individuals with BD [31]. These findings challenge the rigid boundaries of traditional diagnoses. The similar network structures observed across affective disorder subgroups suggest that BPD symptoms follows a dimensional organization rather than being confined to specific diagnoses. Network analysis provides a framework to understand this overlap by examining the dynamic interplay between symptoms rather than relying on categorical classifications [16]. Given these shared vulnerabilities, a network-based perspective may offer a more comprehensive understanding of BPD symptoms and affective disorder.

Although differences in severity were observed, with BD II showing more pronounced BPD symptoms (Table 2), the lack of significant differences in network structures across clinical subgroups (MDD, BD I, and BD II) suggests that affective disorders share core symptoms. Our NCT findings further indicate that, despite diagnostic differences, subjects with affective disorders exhibit a distinct network structure compared to those in the control group. This suggests that BPD symptoms, such as emotional instability and impulsive behavior, follow a similar organization across diagnostic categories, supporting a transdiagnostic perspective [33, 34]. These findings suggest that subjects with affective disorders share a common underlying structure of BPD symptom interconnections, which may contribute to symptom persistence and severity. The observed structural divergence between clinical and control groups highlights the potential influence of affective disorders on BPD symptoms network organization, reinforcing the need for targeted interventions. Thus, the absence of significant differences in network structures across diagnostic groups may account for the variability in symptom severity and comorbidity, reinforcing the idea that these disorders share common traits and mechanisms rather than being distinct entities. These findings suggest that a unified therapeutic approach aimed at disrupting maladaptive symptom connectivity may be beneficial across clinical populations.

Disparities between our findings and those of previous research may be attributed to several key factors. First, this study specifically focused on subjects with affective disorders and BPD symptoms. The distinct clinical profiles and comorbidities in our sample influenced the organization of BPD symptoms in the network, providing a comprehensive view of their interplay. Previous research has often investigated BPD symptoms in general populations or individuals with a BPD diagnosis, overlooking its unique symptom organization in those with affective disorders [1, 5]. Incorporating a control group without psychiatric disorders highlights both shared and distinct features of BPD symptoms across different populations. Second, methodological differences are also important. Research favors factor analysis [35, 36], aiming to identify a single latent factor for BPD symptoms [30]. In contrast, network analysis was used in our study to explore the direct interactions among symptoms [10, 16]. This shift in the analytical perspective revealed five distinct symptom communities, offering a more detailed and dynamic representation of BPD symptoms. Network analysis revealed that “feel empty” was the most central symptom across all affective disorder subgroups, emphasizing its transdiagnostic significance–an insight traditional factor analysis may overlook [37, 38]. Additionally, the robustness of the network analysis allowed us to capture the complex relationships within the BPD symptoms system that factor analysis may overlook. This approach provides new insights into BPD symptoms symptomatology, highlighting its multifaceted nature and intricate component relationships [4, 29]. Although previous research reveal emotional instability and impulsivity as core BPD symptoms, our findings suggest that BPD symptoms consists of multiple interrelated symptom communities, including emotional instability, anger dysregulation and self-harm, interpersonal difficulties and emptiness, impulsivity, and reverse-scored positive traits. This structural model provides a more comprehensive and insightful understanding of BPD symptoms symptomatology, exceeding the level of detail provided by broad diagnostic categories. Finally, our study also examined differences across affective disorder subgroups. Although research highlights the high comorbidity between BPD and affective disorders [5, 7], few studies systematically compared BPD symptom networks across MDD, BD I, and BD II. Our findings indicate that despite heightened severity in BD II, the overall structure and interconnections of BPD symptoms remained consistent across clinical groups. This finding underscores the potential transdiagnostic nature of BPD symptoms within affective disorders and highlights the value of network analysis in capturing these complex relationships.

Several limitations observed in this study should be addressed in future investigations. First, there are certain limitations related to participant selection. In the participant group, individuals with comorbid conditions such as BPD and participant in the active phase of symptoms were not excluded, and the severity and remission of mood symptoms were not assessed. Furthermore, this study relies solely on self-reported absence of psychiatric history for control group recruitment, without verification through structured clinical interviews by mental health professionals. Future studies should incorporate structured diagnostic interviews conducted by trained clinicians to enhance the accuracy of healthy control selection. Second, as PAI-BOR—a self-report instrument—was employed in this study for network analysis, potential biases inherent in self-reports must be considered, particularly among individuals exhibiting high borderline personality disorder symptoms. Some individuals may unintentionally overestimate their symptoms due to heightened emotional sensitivity or cognitive distortions, while others may underreport them by minimizing or concealing distress. Moreover, statistical analysis excludes clinical characteristics such as treatment duration or medication and environmental factors such as COVID-19, requiring cautious interpretation. Third, it employed a cross-sectional design, signifying that the data were obtained at a single specific time point. Such a design limits our ability to establish causal relationships among BPD symptoms. However, while it is plausible that the central symptom influences adjacent BPD symptoms or vice versa, it is also possible that the symptoms are interconnected and mutually reinforce each other [39]. Additionally, a certain limitation of our study is the failure to investigate the directional aspects of these relationships. Future research using a longitudinal design is thus warranted to elucidate the causal directions of these relationships. Fourth, the study participants were exclusively diagnosed with affective disorders, especially MDD, BD I, and BD II. This focused approach limits the applicability of the findings to these conditions, thereby limiting their generalizability to the broader spectrum of mood and other mental disorders. This specificity limits the generalizability of the findings to broader populations like individuals with BPD symptoms without an accompanying affective disorder or those exhibiting BPD symptoms alongside other concurrent mental conditions. Moreover, the imbalance in the representation of BD II (BD II; n = 418) compared to MDD (MDD; n = 245) and BD I (BD I; n = 120) within the present sample may introduce selection bias, as the core BPD symptoms symptomatology can vary between these diagnostic subgroups. Given the dissimilar core BPD symptoms symptomatology among distinct diagnostic subgroups, it becomes evident that forthcoming investigations warrant a more diverse participant cohort and equitably distributed representation ratios.

Despite these limitations, this study represents a significant advancement in understanding of the network structure of BPD symptoms. The insights gained from this study highlight the intricate relationships among BPD symptoms, laying the groundwork for the creation of precise and effective interventions for individuals with BPD. These limitations underscore the complexity of the subject matter and highlight the areas of focus for future research.

## 5. Conclusion

The findings of this study provide valuable insights into the role of BPD symptoms in affective disorders. The symptom of “Feel empty” plays a crucial role in BPD symptoms, and there are five distinct BPD symptoms communities. These findings could be used to improve our understanding of this disorder, and further develop more effective treatment interventions, and identify individuals at risk of developing BPD symptoms.

## Availability of Data and Materials

The datasets generated and/or analyzed during the current study are available from the corresponding author on reasonable request.

## Supplementary Material

Supplementary material associated with this article can be found, in the online version, at https://doi.org/10.31083/AP47292.

## References

[1] Grant BF, Chou SP, Goldstein RB, Huang B, Stinson FS, Saha TD (2008). Prevalence, correlates, disability, and comorbidity of DSM-IV borderline personality disorder: results from the Wave 2 National Epidemiologic Survey on Alcohol and Related Conditions. *The Journal of Clinical Psychiatry*.

[2] Morey LC (2004). *The Personality Assessment Inventory (PAI)*.

[3] Beatson JA, Rao S (2013). Depression and borderline personality disorder. *The Medical Journal of Australia*.

[4] Köhne ACJ, Isvoranu AM (2021). A Network Perspective on the Comorbidity of Personality Disorders and Mental Disorders: An Illustration of Depression and Borderline Personality Disorder. *Frontiers in Psychology*.

[5] Bayes AJ, McClure G, Fletcher K, Román Ruiz Del Moral YE, Hadzi-Pavlovic D, Stevenson JL (2016). Differentiating the bipolar disorders from borderline personality disorder. *Acta Psychiatrica Scandinavica*.

[6] You JS, Lee CW, Park JY, Jang Y, Yu H, Yoon J (2022). Borderline Personality Pathology in Major Depressive Disorder, Bipolar I and II Disorder, and Its Relationship With Childhood Trauma. *Psychiatry Investigation*.

[7] Zimmerman M, Mattia JI (1999). Axis I diagnostic comorbidity and borderline personality disorder. *Comprehensive Psychiatry*.

[8] Tomko RL, Trull TJ, Wood PK, Sher KJ (2014). Characteristics of borderline personality disorder in a community sample: comorbidity, treatment utilization, and general functioning. *Journal of Personality Disorders*.

[9] Richetin J, Preti E, Costantini G, De Panfilis C (2017). The centrality of affective instability and identity in Borderline Personality Disorder: Evidence from network analysis. *PloS One*.

[10] Demyttenaere K, Anthonis E, Acsai K, Correll CU (2022). Depressive Symptoms and PANSS Symptom Dimensions in Patients With Predominant Negative Symptom Schizophrenia: A Network Analysis. *Frontiers in Psychiatry*.

[11] Sheehan DV, Lecrubier Y, Sheehan KH, Amorim P, Janavs J, Weiller E (1998). The Mini-International Neuropsychiatric Interview (M.I.N.I.): the development and validation of a structured diagnostic psychiatric interview for DSM-IV and ICD-10. *The Journal of Clinical Psychiatry*.

[12] American Psychiatric Association (2013). *Diagnostic and statistical manual of mental disorders: DSM-5*.

[13] von Elm E, Altman DG, Egger M, Pocock SJ, Gøtzsche PC, Vandenbroucke JP (2007). The Strengthening the Reporting of Observational Studies in Epidemiology (STROBE) statement: guidelines for reporting observational studies. *Lancet (London, England)*.

[14] Hong S, Kim Y (1998). A validation study of the Borderline Personality Disorder Scale in Korean university students. *Korean Journal of Clinical Psychology*.

[15] Costantini G, Epskamp S, Borsboom D, Perugini M, Mõttus R, Waldorp LJ (2015). State of the aRt personality research: A tutorial on network analysis of personality data in R. *Journal of Research in Personality*.

[16] Borsboom D, Cramer AOJ (2013). Network analysis: an integrative approach to the structure of psychopathology. *Annual Review of Clinical Psychology*.

[17] Friedman J, Hastie T, Tibshirani R (2008). Sparse inverse covariance estimation with the graphical lasso. *Biostatistics (Oxford, England)*.

[18] Epskamp S, Borsboom D, Fried EI (2018). Estimating psychological networks and their accuracy: A tutorial paper. *Behavior Research Methods*.

[19] Chen J, Chen Z (2008). Extended Bayesian information criteria for model selection with large model spaces. *Biometrika*.

[20] Christensen A, Golino H (2019). Estimating the stability of the number of factors via Bootstrap Exploratory Graph Analysis: A tutorial. *PsyArXiv*.

[21] Golino HF, Epskamp S (2017). Exploratory graph analysis: A new approach for estimating the number of dimensions in psychological research. *PloS One*.

[22] Golino HF, Demetriou A (2017). Estimating the dimensionality of intelligence like data using Exploratory Graph Analysis. *Intelligence*.

[23] Blondel VD, Guillaume JL, Lambiotte R, Lefebvre E (2008). Fast unfolding of communities in large networks. *Journal of Statistical Mechanics: Theory and Experiment*.

[24] Christensen AP, Golino H (2021). Estimating the stability of psychological dimensions via bootstrap exploratory graph analysis: A Monte Carlo simulation and tutorial. *Psych*.

[25] Golino H, Christensen A, Moulder R, Garrido LE, Jamison L, Shi D (2020). EGAnet: Exploratory Graph Analysis-A framework for estimating the number of dimensions in multivariate data using network psychometrics. https://CRAN.R-project.org/package=EGAnet.

[26] McNally RJ (2016). Can network analysis transform psychopathology?. *Behaviour Research and Therapy*.

[27] Epskamp S, Cramer AO, Waldorp LJ, Schmittmann VD, Borsboom D (2012). qgraph: Network visualizations of relationships in psychometric data. *Journal of Statistical Software*.

[28] van Borkulo CD, van Bork R, Boschloo L, Kossakowski JJ, Tio P, Schoevers RA (2023). Comparing network structures on three aspects: A permutation test. *Psychological Methods*.

[29] Vervaet M, Puttevils L, Hoekstra RHA, Fried E, Vanderhasselt MA (2021). Transdiagnostic vulnerability factors in eating disorders: A network analysis. *European Eating Disorders Review: the Journal of the Eating Disorders Association*.

[30] Brown TA, Moore MT (2012). Confirmatory factor analysis. *Handbook of Structural Equation Modeling*.

[31] Frías Á, Baltasar I, Birmaher B (2016). Comorbidity between bipolar disorder and borderline personality disorder: Prevalence, explanatory theories, and clinical impact. *Journal of Affective Disorders*.

[32] Angst J, Marneros A, Angst J (2000). Temperament and personality types in bipolar patients a historical review. *Bipolar disorders: 100 years after manic-depressive insanity*.

[33] Henry C, Mitropoulou V, New AS, Koenigsberg HW, Silverman J, Siever LJ (2001). Affective instability and impulsivity in borderline personality and bipolar II disorders: similarities and differences. *Journal of Psychiatric Research*.

[34] Koenigsberg HW, Harvey PD, Mitropoulou V, New AS, Goodman M, Silverman J (2001). Are the interpersonal and identity disturbances in the borderline personality disorder criteria linked to the traits of affective instability and impulsivity?. *Journal of Personality Disorders*.

[35] Jackson KM, Trull TJ (2001). The factor structure of the Personality Assessment Inventory-Borderline Features (PAI-BOR) Scale in a nonclinical sample. *Journal of Personality Disorders*.

[36] Gardner K, Qualter P (2009). Reliability and validity of three screening measures of borderline personality disorder in a nonclinical population. *Personality and Individual Differences*.

[37] Miller CE, Townsend ML, Day NJS, Grenyer BFS (2020). Measuring the shadows: A systematic review of chronic emptiness in borderline personality disorder. *PloS One*.

[38] Miller CE, Townsend ML, Grenyer BFS (2021). Understanding chronic feelings of emptiness in borderline personality disorder: a qualitative study. *Borderline Personality Disorder and Emotion Dysregulation*.

[39] Bos FM, Snippe E, de Vos S, Hartmann JA, Simons CJP, van der Krieke L (2017). Can We Jump from Cross-Sectional to Dynamic Interpretations of Networks? Implications for the Network Perspective in Psychiatry. *Psychotherapy and Psychosomatics*.

