# Supplementary material for: Exploring Symptoms of Borderline Personality Disorder in People With Affective Disorders: A Network Analysis
Source: Alpha Psychiatry. 2025 Oct 13;26(5):47292. doi: 10.31083/AP47292 (PMC12593768; doi:10.31083/AP47292)
Supplement: Supplementary file 1 [file 2757-8038-26-5-47292-s1.zip › Supplementary material.docx]

Supplementary Material

**Supplementary Fig. 1.** Estimation of the number of dimensions for PAI-BOR 24-symptom-items in affective disorder (N= 783) by exploratory graph analysis.


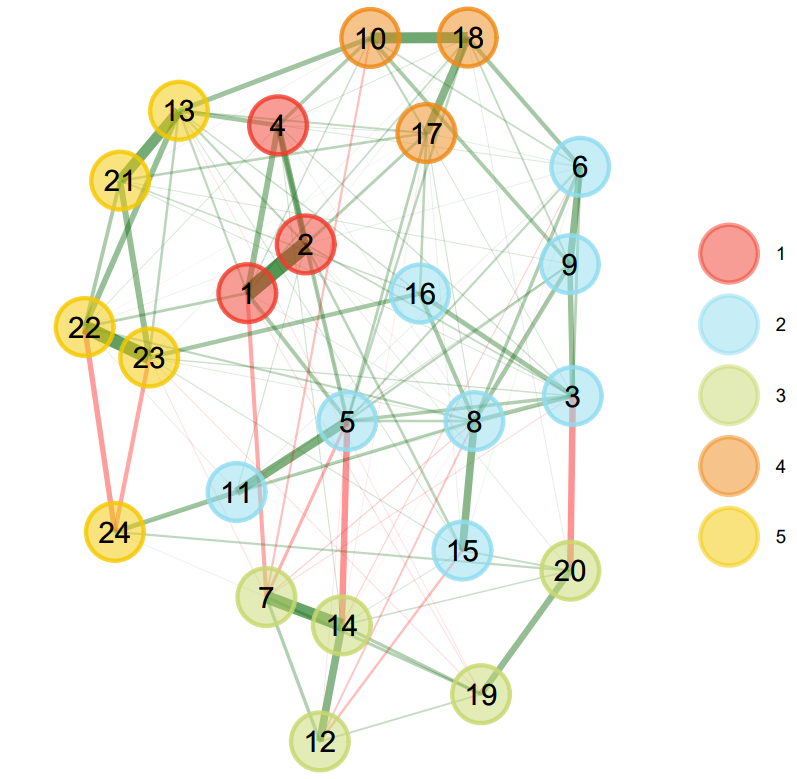


**Supplementary Fig. 2.** Replication of the PAI-BOR 24-symptom-items for five dimensions in affective disorder (N=783) by the Bootstrapped exploratory graph analysis.


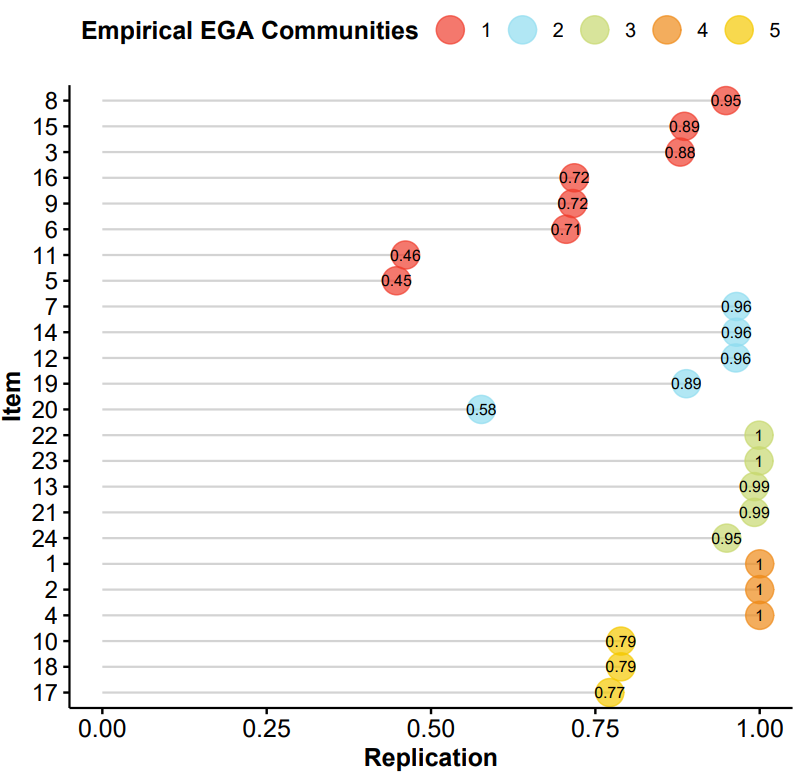


**Supplementary Fig. 3.** Network of the PAI-BOR 24-symptom-items in control group (N= 540)


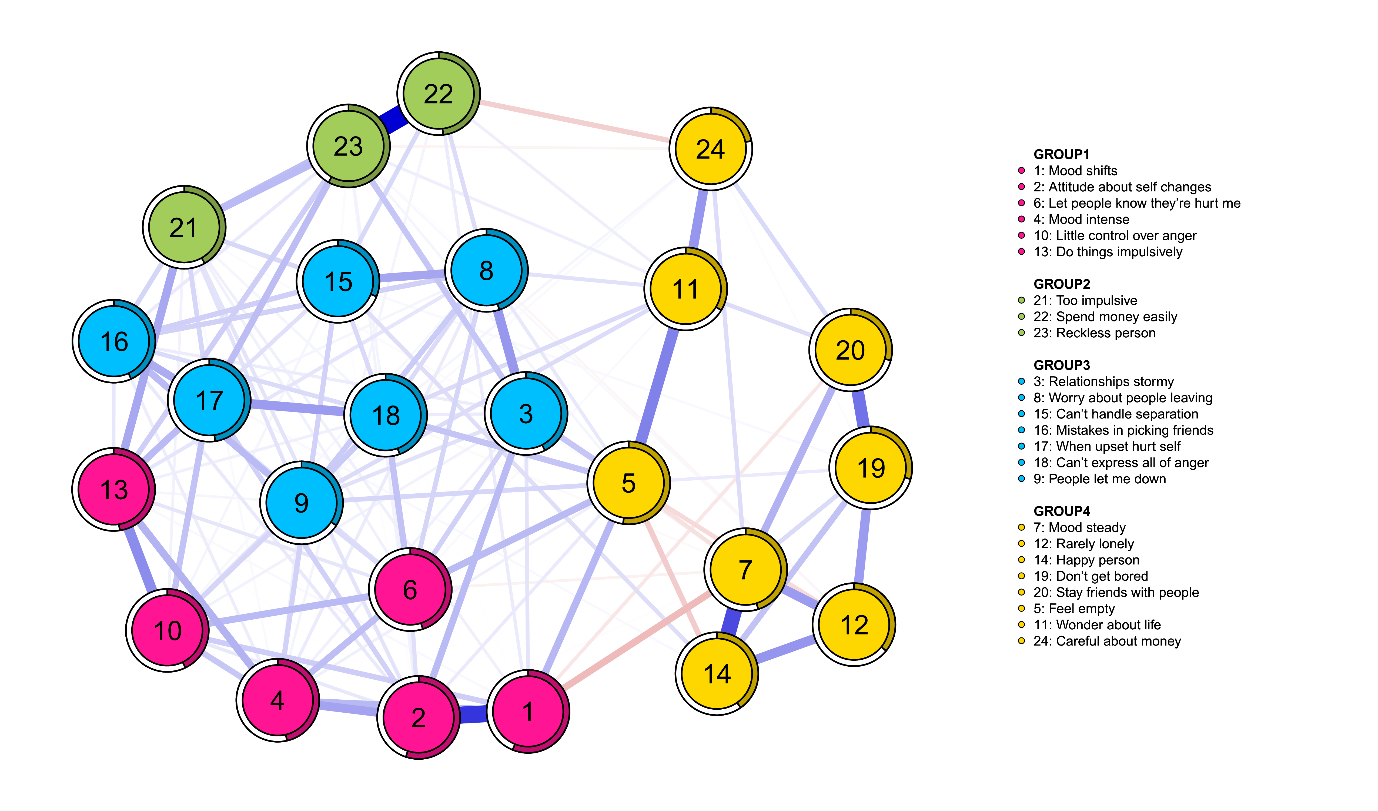


**Supplementary Fig. 4.** Estimation of the number of dimensions for PAI-BOR 24-symptom-items in control group (N= 540) by exploratory graph analysis.

**
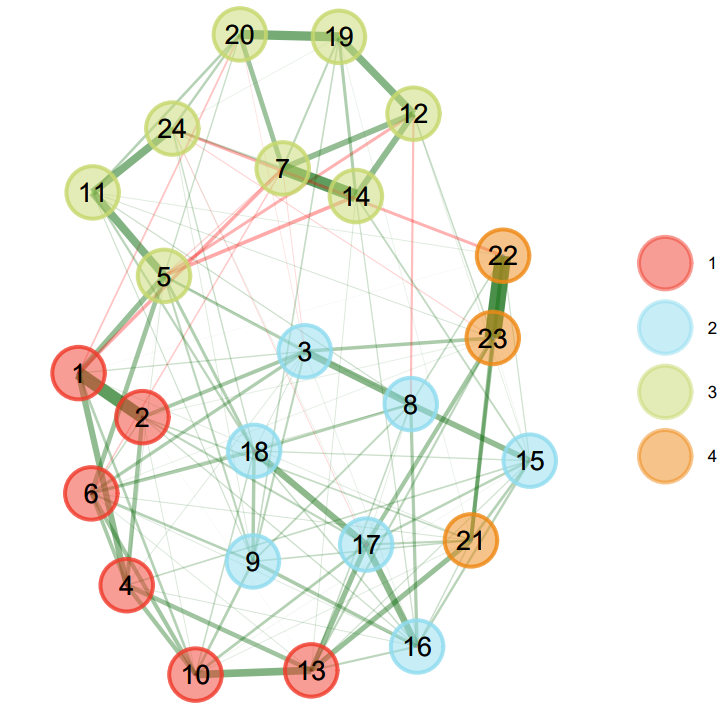
**

**Supplementary Fig. 5.** Bootstrapped difference tests between nodes that were non-zero in the PAI-BOR 24-symptom-items network among 783 subjects with affective disorders.

Note. Gray boxes indicate that the nodes are not significantly different from each other, while black boxes indicate that the nodes are significantly different from each other (*p* < .05).


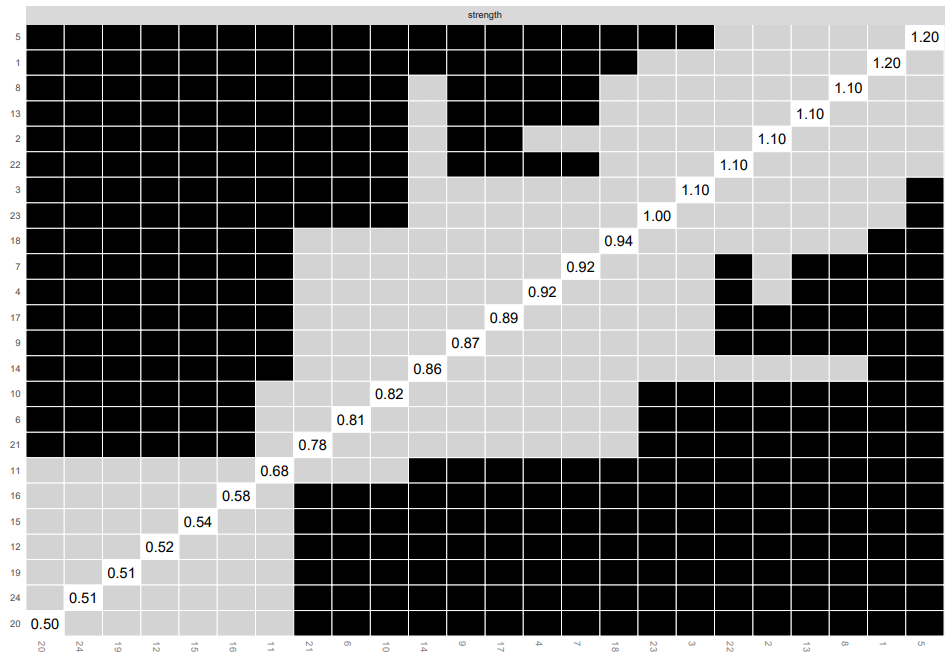


**Supplementary Fig. 6.** Bootstrapped difference tests between nodes that were non-zero in the PAI-BOR 24-symptom-items network among 245 subjects with major depressive disorder.

Note. Gray boxes indicate that the nodes are not significantly different from each other, while black boxes indicate that the nodes are significantly different from each other (*p* < .05).

**
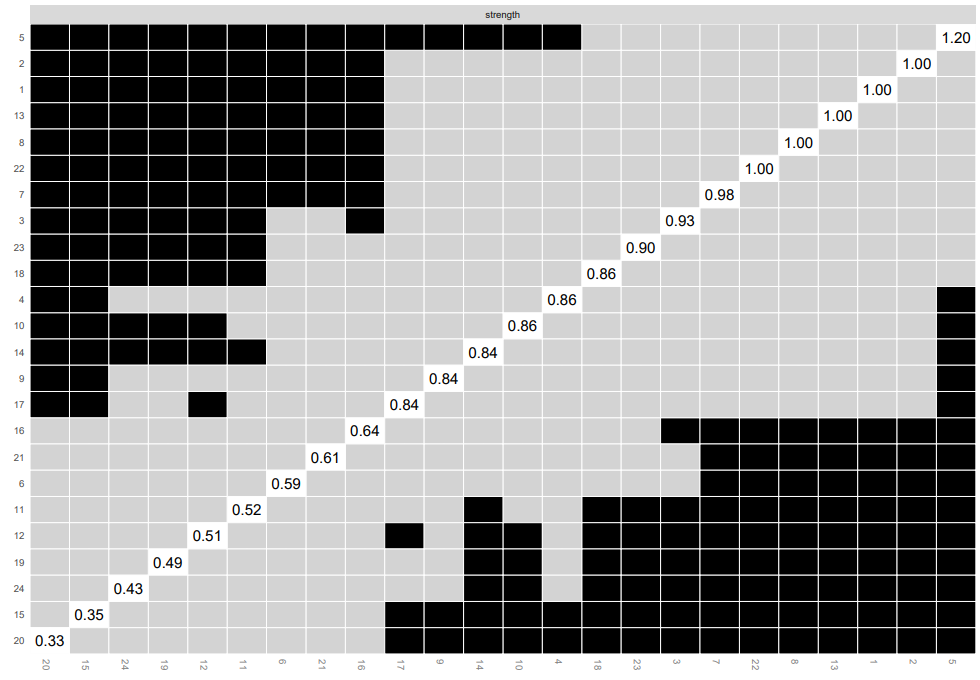
**

**Supplementary Fig. 7.** Bootstrapped difference tests between nodes that were non-zero in the PAI-BOR 24-symptom-items network among 120 subjects with bipolar disorder Ⅰ.

Note. Gray boxes indicate that the nodes are not significantly different from each other, while black boxes indicate that the nodes are significantly different from each other (*p* < .05).

**
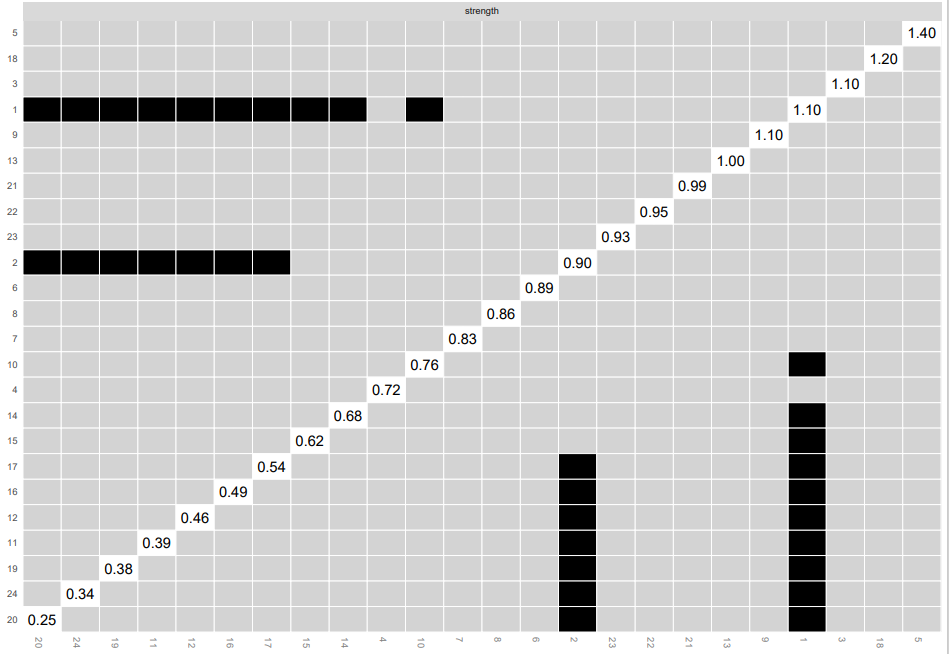
**

**Supplementary Fig. 8.** Bootstrapped difference tests between nodes that were non-zero in the PAI-BOR 24-symptom-items network among 418 subjects with bipolar disorder Ⅱ.

Note. Gray boxes indicate that the nodes are not significantly different from each other, while black boxes indicate that the nodes are significantly different from each other (*p* < .05).

**
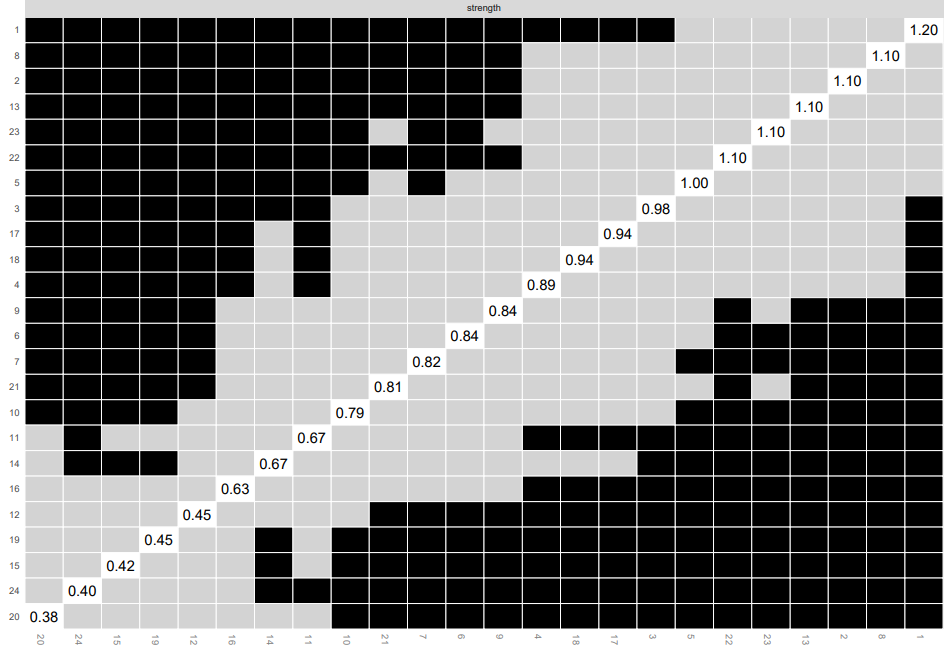
**

**Supplementary Fig. 9.** Bootstrapped difference tests between nodes that were non-zero in the PAI-BOR 24-symptom-items network among 540 normal group.

Note. Gray boxes indicate that the nodes are not significantly different from each other, while black boxes indicate that the nodes are significantly different from each other (*p* < .05).

**
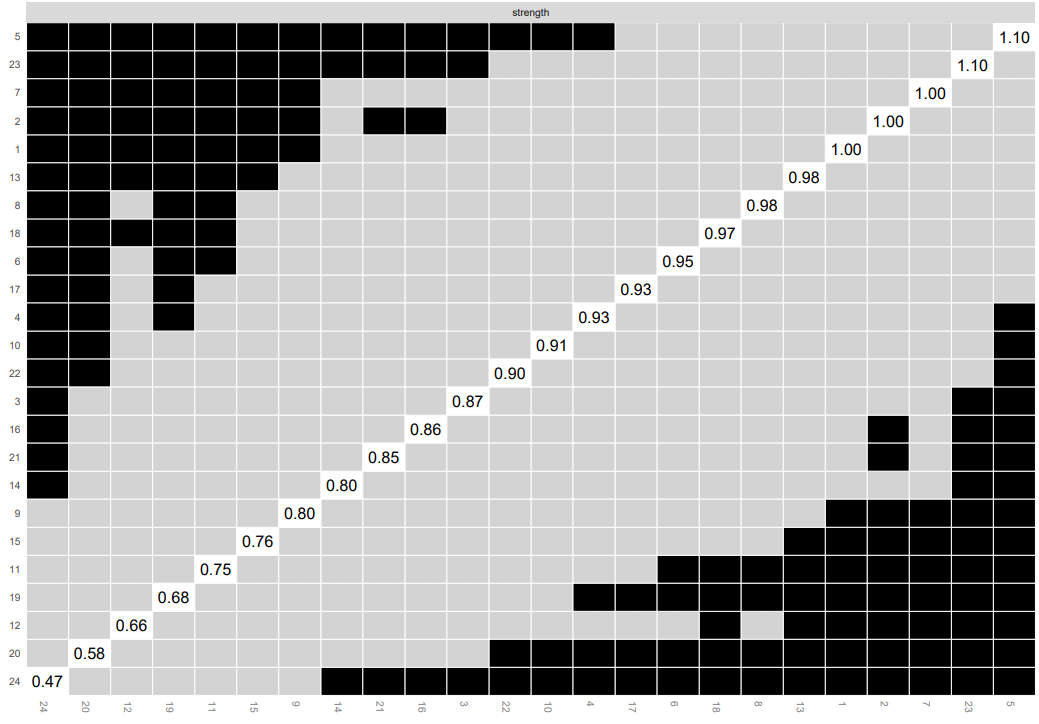
**

**Supplementary Fig. 10.** Bootstrapped the centrality stability of the PAI-BOR 24-symptom-items network among 783 subjects with affective disorder.

Note. The x-axis represents the percentage of cases of the original sample. The y-axis represents the average of correlations between centrality indices in the original sample and sample with case-dropped.

**
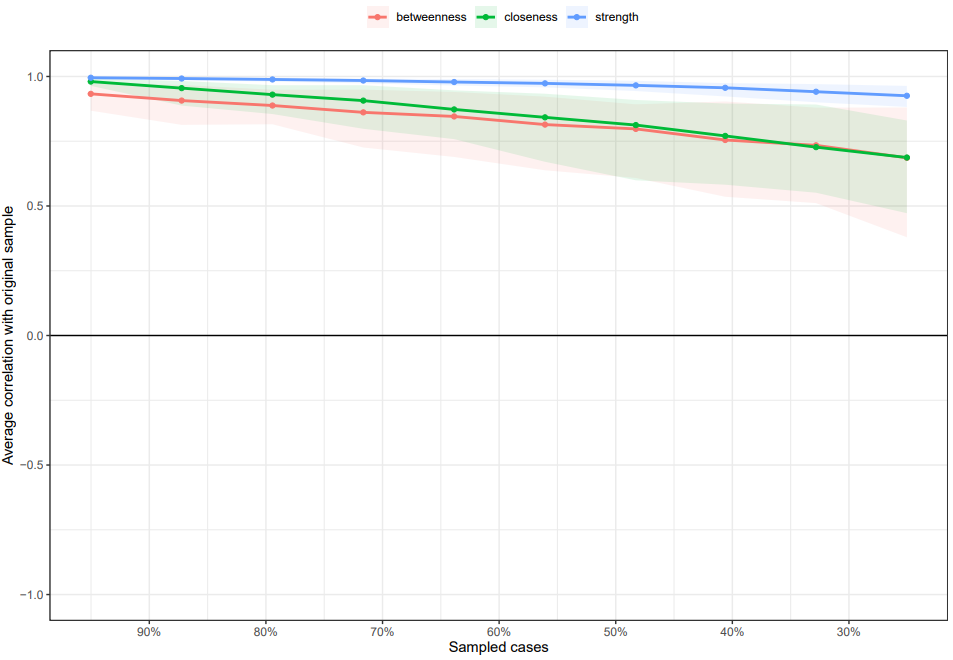
**

**Supplementary Fig. 11.** Edge weight accuracy in the PAI-BOR 24-symptom-items network

Note. Bootstrapped (nBoot = 1,000) confidence intervals (CIs) of the edge weights in the 24-item BPD network. The red line indicates the edge weights values and the gray area the 95% CIs. Each y-axis represents one edge of the network, ordered from the edge with the highest edge-weight to the edge with the lowest edge-weight.

**
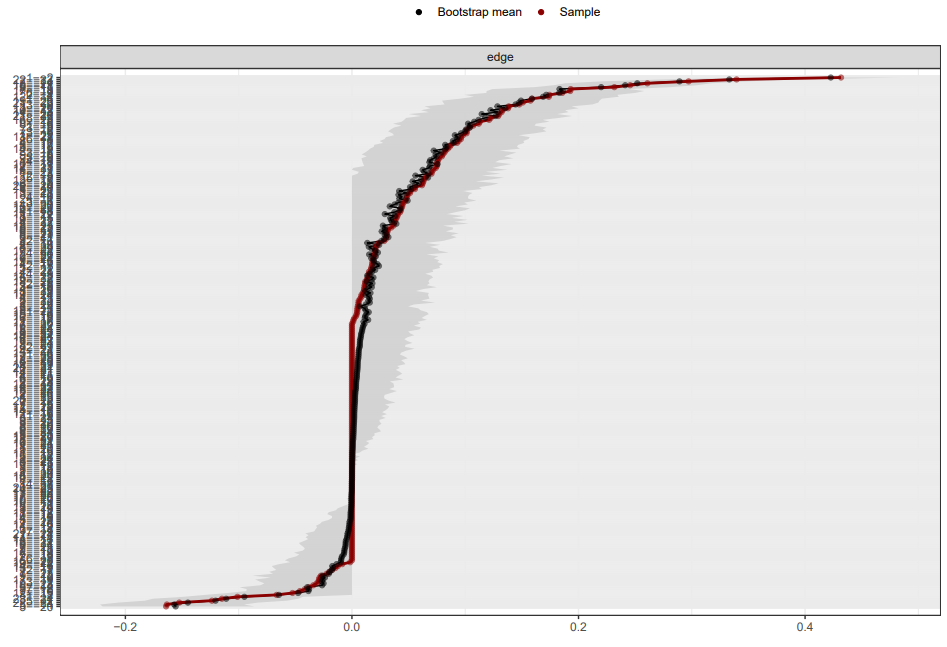
**

**Supplementary Fig. 12.** Bootstrapped difference tests between edge-weights that were non-zero in the PAI-BOR 24-symptom-items network among 783 subjects with affective disorder.

Note. Gray boxes indicate that the nodes are not significantly different from each other, while black boxes indicate that the nodes are significantly different from each other (*p* < .05).

**
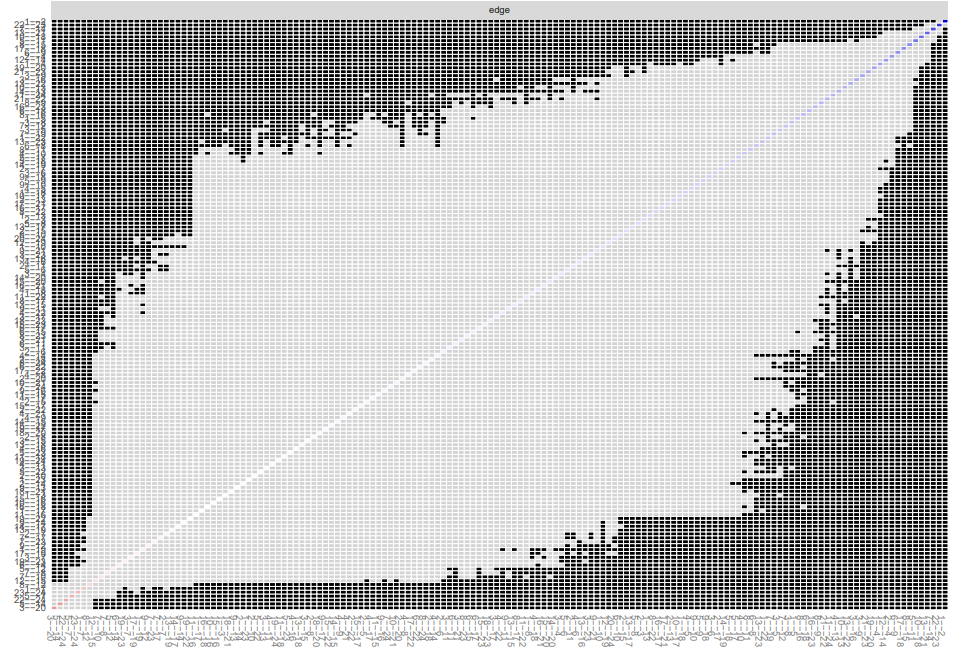
**
